# An *In Vitro* Approach to Study Effects of Prebiotics and Probiotics on the Faecal Microbiota and Selected Immune Parameters Relevant to the Elderly

**DOI:** 10.1371/journal.pone.0162604

**Published:** 2016-09-09

**Authors:** Yue Liu, Glenn R. Gibson, Gemma E. Walton

**Affiliations:** Department of Food and Nutritional Sciences, Whiteknights, PO Box 226, University of Reading, Reading, United Kingdom; Southern Illinois University School of Medicine, UNITED STATES

## Abstract

The aging process leads to alterations of gut microbiota and modifications to the immune response, such changes may be associated with increased disease risk. Prebiotics and probiotics can modulate microbiome changes induced by aging; however, their effects have not been directly compared. The aim of this study was to use anaerobic batch culture fermenters to assess the impact of various fermentable carbohydrates and microorganisms on the gut microbiota and selected immune markers. Elderly volunteers were used as donors for these experiments to enable relevance to an aging population. The impact of fermentation supernatants on immune markers relevant to the elderly were assessed *in vitro*. Levels of IL-1β, IL-6, IL-8, IL-10 and TNF-α in peripheral blood mononuclear cell culture supernatants were measured using flow cytometry. *Trans*-galactooligosaccharides (B-GOS) and inulin both stimulated bifidobacteria compared to other treatments (p<0.05). Fermentation supernatants taken from faecal batch cultures supplemented with B-GOS, inulin, *B*. *bifidum*, *L*. *acidophilus* and *Ba*. *coagulans* inhibited LPS induced TNF-α (p<0.05). IL-10 production, induced by LPS, was enhanced by fermentation supernatants from faecal batch cultures supplemented with B-GOS, inulin, *B*. *bifidum*, *L*. *acidophilus*, *Ba*. *coagulans* and *Bac*. *thetaiotaomicron* (p<0.05). To conclude, prebiotics and probiotics could lead to potentially beneficial effects to host health by targeting specific bacterial groups, increasing saccharolytic fermentation and decreasing inflammation associated with aging. Compared to probiotics, prebiotics led to greater microbiota modulation at the genus level within the fermenters.

## Introduction

Currently, there is an increase in life expectancy, thus a rapidly aging population. According to WHO, the population of adults aged 60 and over has doubled since 1980, and by 2050 this figure is forecast to reach 2 billion, outnumbering children under 14 years of age [1]. The aging population has several health issues, these may include reduced organ function and compromised immune system. Intestinal motility and transit time are slow in older people; this can lead to faecal impaction and constipation [2–4]. Slow colonic transit can also bring about increases in detrimental metabolites of proteolytic bacteria, such as ammonia and amines [5]. There are also problems associated with the diet of elderly people, for example, more limited foods, incorporating less carbohydrates and fewer nutrients [5]. This may be a result of higher thresholds for taste and smell than younger adults [6] and loss of tooth function with difficulties in masticating [7] and swallowing [8].

Elderly populations have a depleted immune defence to exogenous infectious agents but may experience increased immune response to endogenous signals caused by damage of host cells and tissues [9]. This process is loosely termed immunosenescence [10]. Increased levels of cytokines, such as interleukin-6 (IL-6), IL-1β, and tumour necrosis factor-α (TNF-α), decreased phagocytosis and natural killer (NK) cell activity have been observed in elderly populations [11–13]. During aging, the clearance of apoptotic cells is impaired and incomplete [14]. As such, abnormal immune responses including autoimmunity are observed during immunosenescence. In addition, naive B cells generated by bone marrow decrease with increasing age [15], resulting in a reduced ability to protect the host against infectious agents.

There are a great variety of microorganisms inhabiting the human intestinal tract, which is important in maintaining host health and providing a natural defence against invading pathogens [16, 17]. Due to age-related changes in the gastrointestinal tract, such as decreased transit time and increased mucosal membrane permeability [18], as well as changes in diet and immune function, microbial dysbiosis may occur in elderly populations [13]. Studies have shown decreased viable counts of *Bacteroides* in the elderly compared to younger adults [19, 20]. A reduction of bifidobacteria in terms of numbers and species diversity is also a notable change in elderly populations [19–25]. An increase in facultative anaerobes, such as streptococci, enterococci and enterobacteria is a confirmed age-related phenomenon [20, 21, 23, 26–28].

Overall changes in microbial numbers and species diversity may lead to a reduction in gut function which impacts on the immune response, and potentially results in greater susceptibility to gastrointestinal disorder and metabolic syndrome [24, 28]. In addition, aging is associated with declined mucin production which may lead to increased gut barrier permeability and may enable resident microbiota to more easily traverse gut epithelium cells [29]. A damaged mucosal barrier function with changes in the gut microbiota in elderly people may therefore increase translocation of pathogens and susceptibility to infection [29]. Consequently, these will lead to immune dysregulation. The triadic relationship between an impaired gastrointestinal tract, imbalanced gut microbiota and inflammation has been associated with disease risk in elderly populations, such as infections, colorectal cancer [29] and *Clostridium difficile* associated diarrhoea [30].

The aging population are prone to infections; therefore, there should be heightened attention to their physiological welfare. Several *in vitro* and *in vivo* approaches have shown that prebiotics and probiotics can modulate the gut microbial composition towards a potentially healthier community structure in the elderly [2, 13, 31–33]. They have also been shown to improve immune function in elderly persons [13, 34–36]. A dietary prebiotic is ‘a selectively fermented ingredient that results in specific changes, in the composition and/or activity of the gastrointestinal microbiota, thus conferring benefit(s) upon host health’ [37]. Probiotics are ‘live microorganisms that, when administered in adequate amounts, confer a health benefit on the host’ [38, 39].

The prebiotics B-GOS and inulin have been shown to modulate microbiota composition in elderly persons [2, 13]. B-GOS has also been found to enhance immune function [13]. Compared to inulin, short chain FOS has also been shown to improve immune function in older persons [34, 40, 41]. Two synbiotics containing mixtures of *Bifidobacterium bifidum* BB-02, *Bifidobacterium lactis* BL-01 and inulin, and a mixture of *Lactobacillus acidophilus* and lactitol, were shown to exert positive effects on microbiota composition in healthy elderly persons [16, 42]. *Bacillus coagulans* GBI-30, 6086 (GanedenBC^30^ (BC30)) has the potential to suppress the growth of pathogens [43]. In an *in vitro* study, both the cell wall and the metabolite fractions of BC30 were shown to possess immune modulation properties, anti-inflammatory effects and direct induction of IL-10 [36].

Few studies have directly compared the efficacy of both probiotics and prebiotics in modulation of gut microbiota composition and immune function within the same setting. By targeting a population aged 60–75 it may be possible to target the microbiota and the immune changes in their early stages. Therefore, the aim of this study was to use an *in vitro* approaches with samples from donors aged 60–75 years to compare the impact of prebiotics and probiotics on the gut microbiota and selected immune markers relevant to the elderly. Common commercial prebiotic and probiotic products were used. Inulin and B-GOS were used as prebiotics; *Bifidobacterium bifidum*, *Lactobacillus acidophilus*, and *Bacillus coagulans* were used as probiotics. Placebos were microcrystalline cellulose and maltodextrin. *Bac*. *thetaiotaomicron* and *S*. *typhimurium* were also used to investigate the influences of commensal bacteria and a pathogen respectively on the test parameters.

## Materials and Methods

### Chemicals

Bacteriological growth medium supplements were obtained from Oxoid Ltd. (Basingstoke, Hants, U.K.). Inulin was obtained from BENEO GmbH (Mannheim, Germany) and B-GOS from Clasado Ltd (Milton Keynes, UK). All nucleotide probes used for fluorescent *in situ* hybridisation (FISH) were commercially synthesised and labelled with the fluorescent dye Cy3 at the 5′ end (Sigma-Aldrich Co. Ltd., Spain). Sterilisation of media and instruments was carried out by autoclaving at 121°C for 15 min.

### Bacterial strains and culture preparation

*Bacillus coagulans*: GBI-30 (PTA-6086, GanedeBC^30^TM) was sourced from American Type Culture Collection (Manassas, United States) and *Bacteroides thetaiotaomicron* NCTC 10582 from Health Protection Agency Culture Collection (Salisbury, UK). For *Bifidobacterium bifidum* NCIMB 30179 (PXN23), *Lactobacillus acidophilus* NCIMB 30179 (PXN23), *Bacteroides thetaiotaomicron* NCTC 10582 and *Salmonella typhimurium* SL134. For each organism growth curves of optical density (OD_660nm_) against colony forming units (CFU) per millilitre were conducted in triplicate by regular sampling of 48 hour cultures. *B*. *bifidum* and *L*. *acidophilus* were grown in de Man—Rogosa—Sharpe (MRS) broth (10 ml) (Oxoid Ltd, Basingstoke, Hampshire, UK), at 37°C to late log phase under anaerobic (10:10:80%; H_2_:CO_2_:N_2_) conditions. After centrifuging at 14 000 g for 10 min, supernatants were removed. According to growth curves and standards, concentrations of cells were adjusted to 5×10^8^ CFU/ml by addition of anaerobic phosphate buffered saline (1 M, pH 7.4). Finally, 1ml of 5×10^8^ CFU/ml of cells was added to batch culture vessels immediately. *S*. *typhimurium* was grown in Luria Bertani (LB) broth (10 ml) (Oxoid Ltd, Basingstoke, Hampshire, UK) to late log phase in a shaking incubator at 37°C. *Bac*. *thetaiotaomicron* was grown in nutrient broth (10 ml) (Oxoid Ltd, Basingstoke, Hampshire, UK) to late log phase anaerobically (10:10:80%; H_2_:CO_2_:N_2_) at 37°C. They were treated in the same way as the probiotics before adding to the batch culture fermenters. 1ml of 5×10^8^ CFU/ml of *Salmonella typhimurium* SL1344 and 1ml of 5×10^8^ CFU/ml of *Bacteroides thetaiotaomicron* NCTC 10582 were added to corresponding batch culture vessels immediately.

*Bacillus coagulans* GBI-30 product contained 1×10^9^ CFU in each capsule. Half a capsule (5×10^8^ CFU) was suspended in 1ml phosphate buffered saline (1 M, pH 7.4). The cells were then immediately added to batch culture vessels.

### Faecal sample preparation

Faecal samples were collected from three individuals (62–66 years of age). All volunteers were in good health and had not ingested antibiotics for at least 6 months before the study. Samples were collected on site on the day of the experiment and were used immediately. These were diluted 1:10 (w/v) with anaerobic phosphate buffered saline (PBS; 0.1 M; pH 7.4) and homogenised in a stomacher for 2 min (460 paddle beats/min). Resulting faecal slurries from each individual were used to inoculate batch culture vessels.

### Faecal batch culture fermentation

Three separate fermentation experiments were carried out. Batch culture fermentation vessels were autoclaved and aseptically filled with 135 ml of basal nutrient medium (peptone water (2 g/l), yeast extract (2 g/l), NaCl (0.1 g/l), K_2_HPO_4_ (0.04 g/l), KH_2_PO_4_ (0.04 g/l), NaHCO_3_ (2 g/l), MgSO_4_·7H_2_O (0.01 g/l), CaCl_2_·6H_2_O (0.01 g/l), tween 80 (2 ml/l), hemin (50 mg/l), vitamin K1 (10 ml/l), L-cysteine (0.5 g/l), bile salts (0.5 g/l), resazurin (1 mg/l)). The vessels were gassed overnight with O_2_-free N_2_ (15 ml/min). Before addition of the faecal slurries, temperature of the basal nutrient medium was set to 37°C by use of a circulating water bath and pH was maintained at 6.8 using a pH controller (Electrolab, UK). The vessels were inoculated with 15 ml of faecal slurry (1:10, w/w), and in order to mimic conditions located in the distal region of the human large intestine the experiment was carried out under anaerobic conditions, 37°C and pH 6.8− 7.0 for a period of 48 h. During this period, samples (10 ml) were collected at six time points (0, 5, 10, 24, 30 and 48 h). Fluorescent *in situ* hybridisation was used for bacterial enumeration and gas chromatography (GC) for organic acid analysis.

### Inoculation of substrate in the batch culture

Batch culture fermentations were conducted using a range of treatments: control (no treatment), *trans*-galactooligosaccharides mixture (manufactured by Clasado Ltd) called BiMuno^®^ (B-GOS, 1.5g), standard inulin (Orafti^®^ ST, Beneo, Tienen, Belgium; 1.5g), microcrystalline cellulose (1.5g), maltodextrin (1.5g), *B*. *bifidum* (5×10^8^ CFU), *L*. *acidophilus* (5×10^8^ CFU), *Ba*. *coagulans* (5×10^8^ CFU); *Bac*. *thetaiotaomicron* (5×10^8^ CFU, commensal bacteria); and *S*. *typhimurium* (5×10^8^ CFU, pathogen). B-GOS and inulin are common commercial prebiotics. *B*. *bifidum*, *L*. *acidophilus* and *Ba*. *coagulans* are common commercial probiotics. Microcrystalline cellulose and maltodextrin were used as placebo treatments compared to prebiotics. *Bac*. *thetaiotaomicron* and *S*. *typhimurium* were also used to investigate the influences of a commensal bacterium and pathogen. In addition, 0.5g potato starch from Sigma-Aldrich Co. Ltd. (UK) was added to each vessel as a fermentable carbon source.

### Sample processing

In preparation for FISH analysis 375 μl batch culture supernatant was taken in duplicate into two tubes of 4°C 1125 μl 4% (w/v) paraformaldehyde solution and fixed at 4°C for 4 hours. After 4 hours, the batch culture supernatant was centrifuged for 5 minutes at 11337 xg (Eppendorf centrifuge minispin, Eppendorf, UK) at room temperature. The supernatant was carefully removed and discarded. The pellet was re-suspended in 1 ml of cold 1×PBS by aspirating carefully using a pipette. Again, the sample was centrifuged for 5 minutes at 11337 xg at room temperature and the supernatant discarded. The sample was washed again in 1 ml cold PBS as above and centrifuged. All supernatant was carefully removed. Finally, the pellet was re-suspended in 150 μl cold 1×PBS and 150 μl ethanol. The sample was mixed by vortexing and then stored at -20°C.

In preparation for SCFA analysis, 1 ml of batch culture supernatant was taken in duplicate and centrifuged for 10 minutes at 11337 xg. The supernatant was stored at -20°C.

For *in vitro* immunoassays, 1 ml of batch culture supernatant was taken in triplicate, centrifuged for 10 minutes at 11337 xg and filtered through a 0.22 μm filter device (Millipore, Schwalbach, Germany). The cell-free supernatant was finally stored at -20°C.

### Bacterial enumeration

Bacterial populations were enumerated using FISH, with oligonucleotide probes targeting specific regions of 16S rRNA. Probes were commercially synthesised and coated with the fluorescent dye Cy3. The probes used were: Ato 291 for *Atopobium* cluster (ATO) [44], Lab 158 for lactobacilli/enterococci (LAB) [45], Bif 164 for bifidobacteria (BIF) [46], Erec 482 for *Eubacterium rectale*–*Clostridium coccoides* group (EREC) [47], Chis 150 for the *Clostridium histolyticum* group (CHIS) [47], Bac 303 for *Bacteroides—Prevotella* spp. (BAC) [48], and EUB 338 mixture consisting of EUB338, EUB338II and EUB338III for total bacteria (Total) [49]. Conditions of hybridisation and washing for individual probes are given in Table 1. Hybridisation of samples was performed as described by Daims, Stoecker [50]. Briefly, the sample was diluted for each probe. 20 μl diluted sample was added to the well of a Teflon- and poly L-lysine-coated 6-well slide (Tekdon Inc, Myakka City, FL). Slides were dried in a desktop plate incubator for 15 minutes at 46–50°C. Then, slides were dehydrated in 50, 80, 96% (v/v) ethanol series for 3 minutes in each solution and then dried for 2 minutes. For probes Lab 158 and Bif 164, 20 μl of lysozyme was added to each well before dehydration in ethanol to increase cell permeability. Then, the hybridisation mixture (0.9 M NaCl, 0.02 M Tris/HCl (pH 8.0), formamide (if required–Table 1), 10% (w/v) sodium dodecyl sulphate, 4.55 ng ml^-1^ probe) was added to each well, and slides placed on a tray, which was sealed and put in a hybridisation oven for 4h at probe specific hybridisation temperature (Table 1). 20 μl nucleic acid stain 4’, 6-diamidino-2- phenylindole (DAPI; 50 ng μl^-1^) was added to the wash buffer, once the hybridisation had completed, slides were placed into wash buffer (0.9 M NaCl, 0.02 M Tris/HCl (pH 8.0), 0.005 M ethylenediaminetetraacetic acid (EDTA) solution (pH 8.0, Table 1), H_2_O) and warmed at the appropriate temperature for each probe (Table 1) for 10–15 minutes. After washing, slides were dipped into ice-cold distilled water for 2–3 seconds and dried by a stream of compressed air. Finally, antifade solution (Dabco) was added to each well, a cover slip applied and slides examined using fluorescent microscopy (Nikon Eclipse E400; Nikon, Tokyo, Japan). The DAPI-stained cells were examined under ultraviolet light, and hybridised cells viewed with the use of a DM510 filter. For each slide, at least 15 random fields of view were counted. The following formula was used to calculate numbers of bacteria: (0.8 × A1 × 6732.42 × 50 × Dilution factor), where A1 is the average count of 15 fields of view, 6732.42 is area of the well divided by the area of the field of view, multiplying by 50 takes the count back to millilitre of sample. Results were expressed as Log_10_ (bacterial numbers per millilitre batch culture fluid).

**Table 1:** Hybridisation and washing conditions for oligonucleotide probes.

Probe name	Sequence (5’ to 3’)	Hybridisation pre-treatment	Formamide (%) in hybridisation buffer	Hybridisation temperature (°C)	Washing temperature (°C)	Reference
Ato 291	GGTCGGTCTCTCAACCC	Lysozyme	0	50	50	[44]
Lab 158	GGTATTAGCAYCTGTTTCCA	Lysozyme	0	50	50	[45]
Bif 164	CATCCGGCATTACCACCC	Lysozyme	0	50	50	[46]
Erec 482	GCTTCTTAGTCARGTACCG	None	0	50	50	[47]
Chis 150	TTATGCGGTATTAATCTYCCTTT	None	0	50	50	[47]
Bac 303	CCAATGTGGGGGACCTT	None	0	46	48	[48]
EUB338*	GCTGCCTCCCGTAGGAGT	None	35	46	48	[49]
EUB338II*	GCAGCCACCCGTAGGTGT	None	35	46	48	[49]
EUB338III*	GCTGCCACCCGTAGGTGT	None	35	46	48	[49]

* These probes are used together in equimolar concentrations (all at 50 ng μl^−1^)

### Organic acid analysis

Organic acid production was determined by GC. Extraction and derivatisation of samples was conducted according to Richardson, Calder [51]. Briefly, samples were defrosted on ice. Each sample was vortexed and 1 ml sample or a standard solution transferred into a labelled 100 mm×16 mm glass tube (Fisher Scientific UK Ltd, Loughborough) with 50 μl of 2-ethylbutyric acid (0.1 M; internal standard). 0.5 ml concentrated HCl and 2 ml diethyl ether were added to each glass tube and samples vortexed for 1 minute. Samples were centrifuged at 2000 xg for 10 minutes (SANYO MSE Mistral 3000i; Sanyo Gallenkap PLC, Middlesex, UK). The diethyl ether (upper) layer of each sample was transferred to a labelled clean glass tube. A second extraction was conducted by adding another 1 ml diethyl ether, followed by vortexing and centrifugation. The diethyl ether layers were pooled. 400 μl of pooled ether extract and 50 μl *N*-(*tert*-butyldimethylsilyl)-*N*-methyltrifluoroacetamide (MTBSTFA) were added into a GC screw-cap vial. Samples were heated at 80°C for 20 minutes and then left at room temperature for 48 hours to allow lactic acid in the samples to completely derivatise.

A 5890 SERIES II Gas Chromatograph (Hewlett Packard, UK) using an Rtx-1 10m×0.18mm column with a 0.20μm coating (Crossbond 100% dimethyl polysiloxane; Restek, Buckinghamshire, UK) was used for analysis of SCFA. Temperatures of injector and detector were 275°C, with the column programmed from 63°C for 3 minutes to 190°C at 10°C min^-1^ and held at 190°C for 3 minutes. Helium was the carrier gas (flow rate 1.2 ml min^-1^; head pressure 90 MPa). A split ratio of 100:1 was used. The SCFA standard was run every 20 samples to update the calibration as necessary. This standard solution contained (mM): sodium formate, 10; acetic acid, 30; propionic acid, 20; isobutyric acid, 5; n-butyric acid, 20; iso-valeric acid, 5; n-valeric acid, 5; sodium lactate, 10; sodium succinate, 20. Peak areas of the standard solution, to which internal standard was added, were used to calculate response factors for each organic acid with respect to the internal standard. Response factor and peak areas within samples were calibrated and calculated using Chemstation B.03.01 (Agilent Technologies, Cheshire, UK). The response factors were calculated using Eq 1. Amount of organic acids in the samples was calculated using Eq 2.

$$Internal\;Response\;Factor=\frac{areaIS\times amountSC}{amountIS\times areaSC}$$ (1)

IS = Internal Standard; SC = Specific Compound of Interest

$$Amount\;of\;Specific\;Compound=\frac{amountIS\times areaSC\times IRFSC}{areaIS}$$ (2)

IS = Internal Standard; SC = Specific Compound of Interest; IRFSC = Internal Response Factor for Specific Compound of Interest

### Preparation of peripheral blood mononuclear cells

Fasted blood samples were taken from six healthy volunteers aged 60–73 years, in sodium heparin vacutainer tubes (Greiner Bio-One Limited, Gloucestershire, United Kingdom). The study was conducted according to guidelines laid down in the Declaration of Helsinki, and all procedures involving human subjects were approved by the Ethics Committee of the University of Reading. The ethics approval number was UREC 14/05. Written informed consent forms were obtained from all subjects. Blood was layered over an equal volume of lympholyte (Cedarlane Laboratories Limited, Burlington, Ontario, Canada) and centrifuged at 930 xg for 15 min at room temperature. Peripheral blood mononuclear cells (PBMCs) were harvested from the interface, washed once with PBS, and then resuspended in Roswell Park Memorial Institute (RPMI) 1640 medium (containing glutamine, Autogen Bioclear Ltd., Wiltshire, UK). These steps were repeated to achieve low contamination of erythrocyte. The pellet was finally resuspended in RPMI 1640 medium and cell numbers counted using trypan blue and a cell counter (Coulter, Fullerton, CA, USA). Cells were adjusted to the required concentration.

### Viability assays

To determine the appropriate supernatant concentration, PBMC viability, at different supernatant concentrations was determined using the trypan blue test. PBMCs, adjusted to 2×10^6^ cells/ml, were incubated in twenty-four-well plates in the presence of RPMI 1640 medium, pure batch culture medium supernatant, 0h and 24h supernatant from *B*. *bifidum* treated and *S*. *typhimurium* treated vessels separately for 24 h at 37°C in an air—CO_2_ (19:1) atmosphere. The tested supernatant amounts of each treatment were 1%, 1.5%, 2%, 3%, 4%, 5% and 10% (v/v) of 2ml (final working volume). At the end of the incubation, cell numbers were counted using trypan blue test. According to the results, 1% (v/v) was appropriate to use for different treatment supernatants.

### Cytokine stimulation and detection

PBMCs, adjusted to 2×10^6^ cells/ml, were incubated in twenty-four-well plates in the presence of 1 mg/ml lipopolysaccharide (LPS; L4516, Sigma-Aldrich Co. Ltd. UK), 1% (v/v) pure batch culture medium, 1 mg/ml LPS with 1% (v/v) pure batch culture medium or 1 mg/ml LPS with 0h, 5h and 24h 1% (v/v) supernatants from ten vessels for 24 h at 37°C in an air—CO_2_ (19:1) atmosphere. At the end of the incubation, cell culture supernatants were collected and stored at -20°C for later analysis of cytokine production. Non-stimulated cultures were used as blank controls.

The production of IL-1β, IL-6, IL-8, IL-10 and TNF-α was measured using BD^™^ Cytometric Bead Array (CBA) Human Soluble Protein Master Buffer Kit (BD Biosciences, Oxford, UK) and corresponding BD^™^ Cytometric Bead Array (CBA) Human Flex Set (BD Biosciences, Oxford, UK) by BD Accuri^™^ C6 flow cytometer according to the manufacturer’s instructions. BD^™^ CBA analysis software FCAP Array v3.0.1 (BD Biosciences, Oxford, UK) was used to perform data analysis.

### Statistical analysis

All statistical tests were performed with the use of SPSS version 18 (SPSS Inc, Chicago, IL). Results are presented as means (n = 3) ± SD.

For bacterial populations and SCFA concentrations, within the same treatment, differences from 0-h value were tested using paired Student’s t test. At the same time point, differences among treatments were analysed by one-way ANOVA. For cytokine production, differences from LPS value were tested using an independent t test. Within the same fermentation treatments, variations from 0-h values were tested using paired Student’s t test. At the same time point, differences among treatments in cytokine production were analysed by one-way ANOVA. Significant differences were determined by *post hoc* Tukey HSD (Honestly Significant Difference) test. A value of *P* <0.05 indicates a significant difference.

## Results

### Enumeration of bacterial populations by FISH

**Fig 1:**
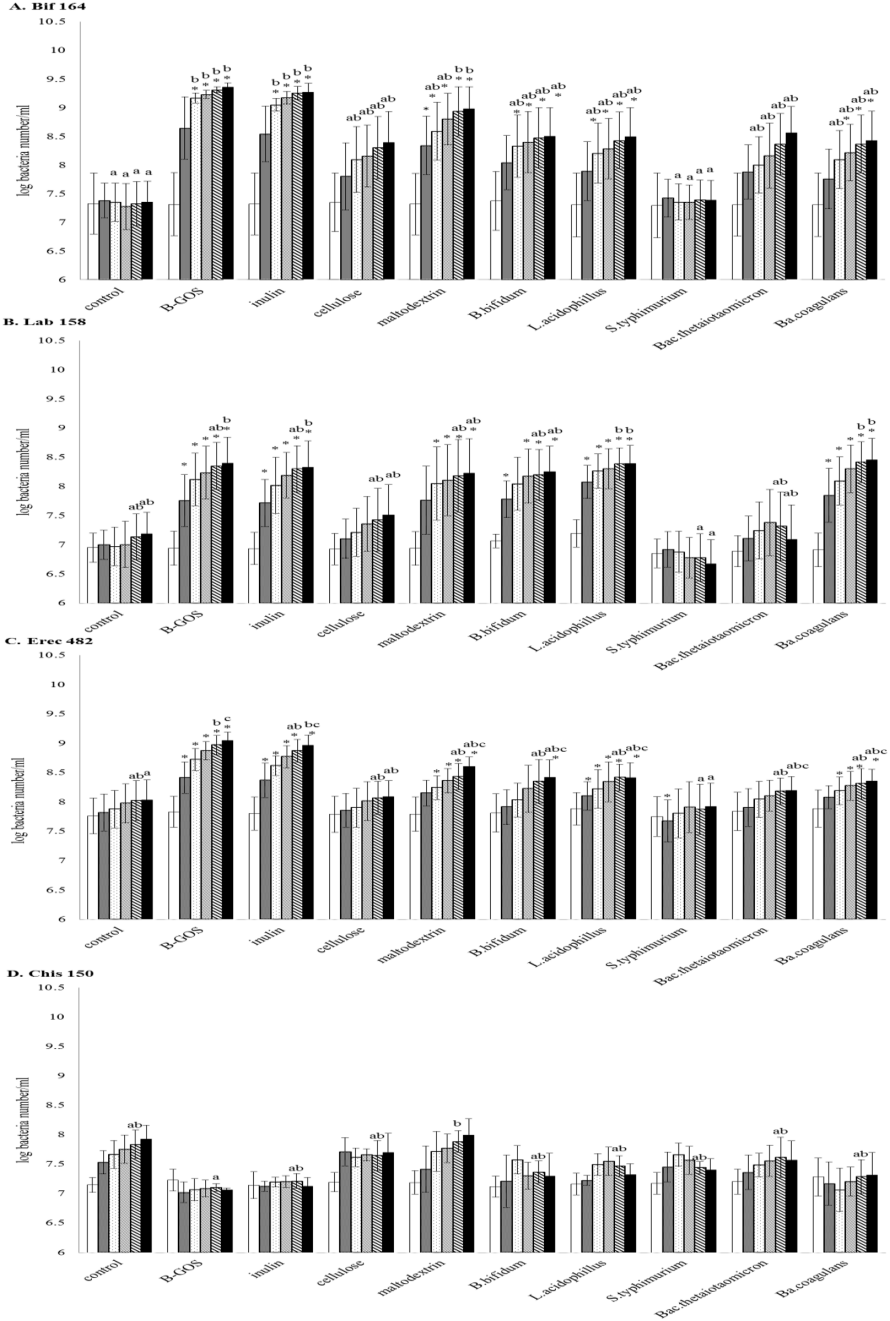
Bacterial populations in pH-controlled batch cultures. Samples were collected at 0 (white), 5 (shaded), 10 (spots), 24 (fine diaganol lines), 30 (spaced diagonal lines) and 48h (black). (A) Bifidobacteria changes during batch culture fermentation. (B) Lactobacilli/enterococci changes during batch culture fermentation. (C) *Eubacterium rectale—Clostridium coccoides* group changes during batch culture fermentation. (D) *Clostridium histolyticum* group changes during batch culture fermentation. Values are mean ± SD from triplicate samples.*, significant differences from the 0h value within the same treatment, p<0.05. Significant differences (p<0.05) among treatments at the same time point are indicated with different letters from the same colour of column.

Bacterial populations are shown in Fig 1 and S1 Fig. In the control vessel, growth of *Atopobium* group (p<0.05, paired Student’s t test) and total bacteria (p<0.05, paired Student’s t test) were stimulated compared to 0h. Growth of bifidobacteria was significantly stimulated by B-GOS, inulin and maltodextrin during fermentations compared to control (p<0.05, ANOVA), with higher levels following B-GOS fermentation. *B*. *bifidum*, *L*. *acidophilus* and *Ba*. *coagulans* were also shown to significantly stimulate bifidobacterial numbers compared to time 0h (p<0.05, paired Student’s t test). Numbers of lactobacilli/enterococci were significantly increased following B-GOS, inulin, *L*. *acidophilus* and *Ba*. *coagulans* at 30h and 48h compared to other treatments (p<0.05, ANOVA). Numbers of *Eubacterium rectale*–*Clostridium coccoides* were increased following B-GOS fermentations at 48h compared to others (p<0.05, ANOVA). In addition, the *Clostridium histolyticum* group was reduced following B-GOS fermentation at 30h compared to other treatments (p<0.05, ANOVA). Following maltodextrin fermentation, levels of *Bacteroides—Prevotella* spp. and *Clostridium histolyticum* group were significantly stimulated compared to other treatments (p<0.05, ANOVA). Following the different treatments, there was no significant change in total bacterial numbers, indicating that overall bacterial numbers remained constant following prebiotic (B-GOS and inulin) and probiotic (*B*. *bifidum*, *L*. *acidophilus* and *Ba*. *coagulans*) use.

### SCFA analysis

**Fig 2:**
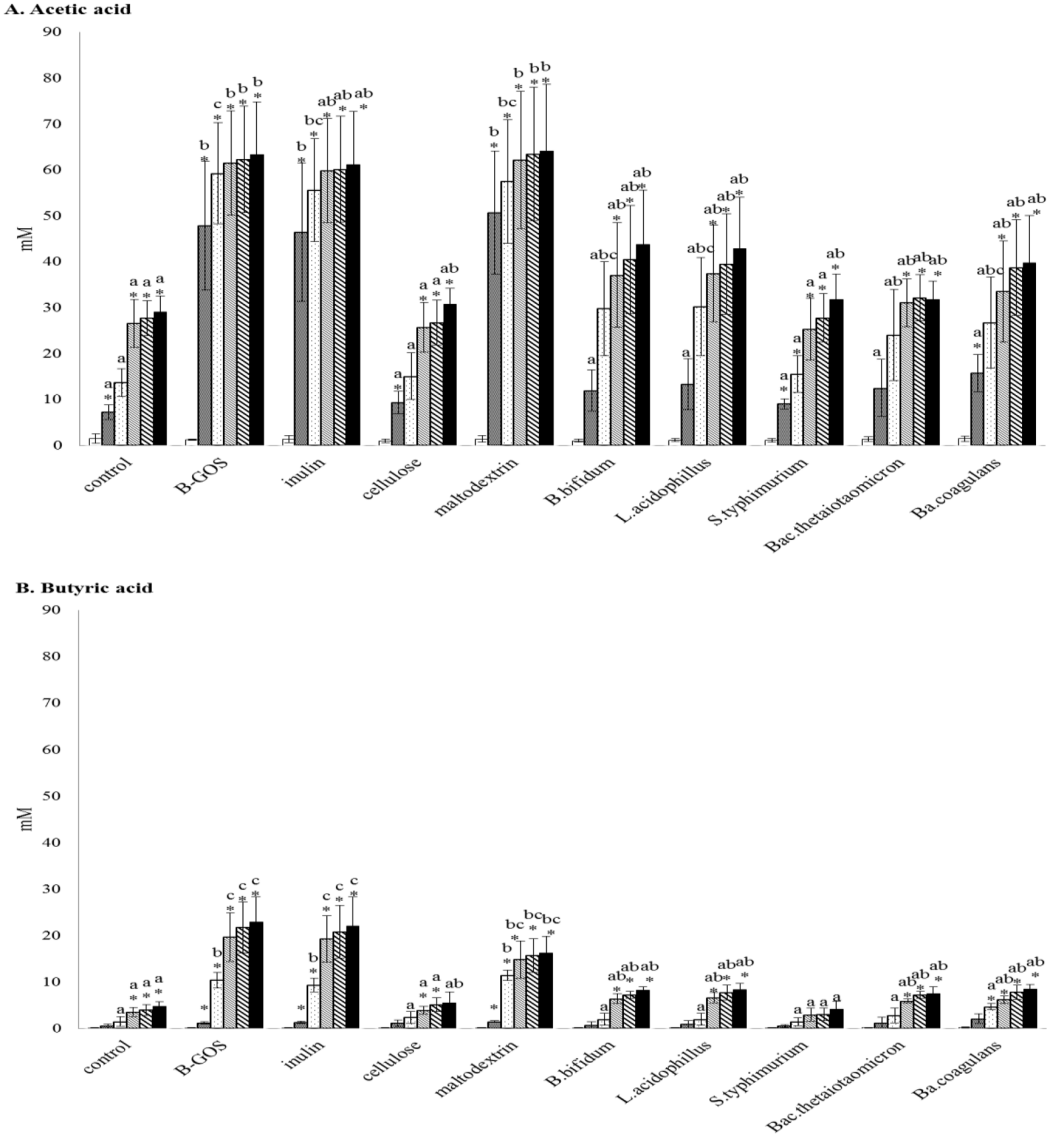
SCFA concentrations in pH-controlled batch cultures. Samples were collected at 0 (white), 5 (shaded), 10 (spots), 24 (fine diaganol lines), 30 (spaced diagonal lines) and 48h (black). (A) Acetate production during batch culture fermentation. (B) Butyrate production during batch culture fermentation. Values are mean ± SD from triplicate samples. *, significant differences from the 0h value within the same treatment, p<0.05. Significant differences (p<0.05) among treatments at the same time point are indicated with different letters from the same colour of column.

Fig 2 and S2 Fig. shows SCFA concentrations during batch culture fermentations. In the control vessel, as a carbon source, potato starch stimulated the production of all SCFAs compared to 0h (p<0.05, paired Student’s t test). Acetate production was significantly stimulated following B-GOS and maltodextrin fermentation compared to other treatments (p<0.05, ANOVA). Propionate production was significantly stimulated following maltodextrin fermentation compared to other treatments (p<0.05, ANOVA). Levels of butyrate were significantly higher in vessels with B-GOS (p<0.05, ANOVA) and inulin (p<0.05, ANOVA) compared to others. Production of branched chain fatty acids, iso-butyrate and iso-valerate, were repressed by prebiotics (B-GOS and inulin) and probiotics (*B*. *bifidum*, *L*. *acidophilus* and *Ba*. *coagulans*) (p<0.05, ANOVA). However, they were significantly higher in vessels with maltodextrin (p<0.05).

### Viability

After 24h incubation of PBMC with supernatants, viability of PBMC cells was measured by trypan blue. Viability was 92% with 1% (v/v) RPMI 1640 medium, 80% with 1%(v/v) pure batch culture medium, 64% and 56% with 1% (v/v) 0h and 24h supernatant from *B*. *bifidum*, 72% with both 1% (v/v) 0h and 24h supernatant from *S*. *typhimurium*. The viability of other amounts (1.5%, 2%, 3%, 4%, 5% and 10% v/v) were all lower than 40%. Differences in viability may have an impact on cytokine production; therefore 1% (v/v) supernatant was used as the most appropriate choice.

### Cytokine production

**Fig 3:**
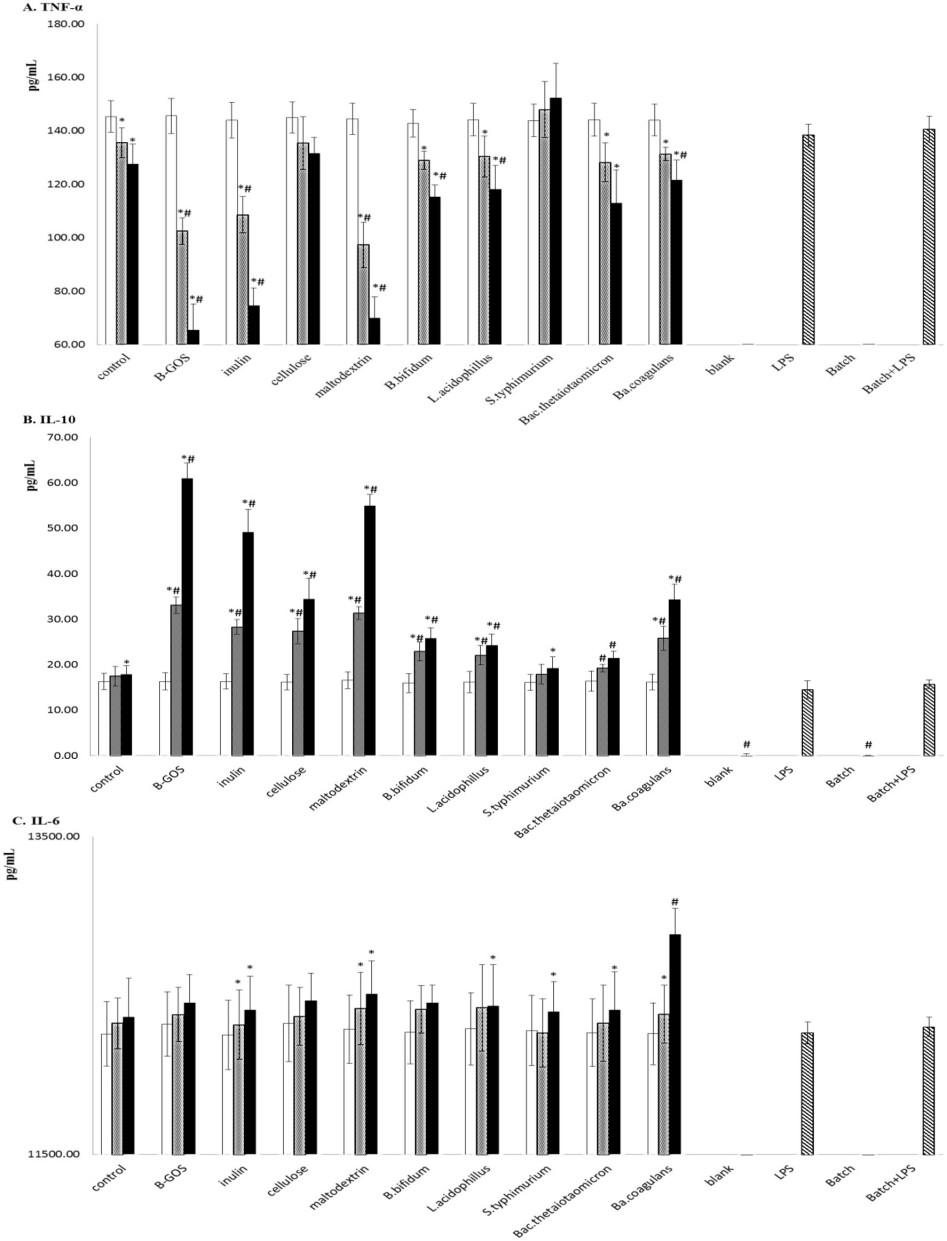
Effect of fermentation supernatants on cytokine production by peripheral blood mononuclear cells (PBMC). Supernatants at 0 (white), 5 (shaded) and 24h (black). Supernatants from PBMCs cultured without batch culture supernatant were used as controls (+/-) (spaced diagonal lines). (A) TNF-α production by PBMC. (B) IL-10 production by PBMC. (C) IL-6 production by PBMC. Values are mean ± SD. PBMC from three volunteers was incubated with batch culture supernatants for 24h. #, significant differences from LPS value p<0.05. *, significant difference from 0-h value within the same fermentation treatments. At the same time point, differences among different treatments in cytokines production were analysed by one-way ANOVA. Significant differences (p<0.05) determined by post hoc Tukey HSD test were not found. In addition, cytokines in non-stimulated PBMC (blank) and in pure batch culture medium-treated PBMC (batch) were also determined. There was no significant difference between them. There was also no significant difference between LPS (LPS-stimulated PBMC) and batch+LPS (PBMC incubated with pure batch culture medium and LPS).

Supernatants from PBMCs cultured without batch culture supernatant were used as controls (+/-). In the absence of LPS, there was no stimulation of IL-1β, IL-6, IL-8, IL-10 and TNF-α (Fig 3 and S3 Fig). Pure batch culture medium did not significantly change the production of IL-1β, IL-6, IL-8, IL-10 and TNF-α induced by LPS (p<0.05, independent t test). LPS-induced TNF-α production, observed in the positive control, was suppressed by 5h and 24h fermentation supernatants from B-GOS, inulin and maltodextrin (p<0.01, independent t test). It was also suppressed by 24h supernatants from *B*. *bifidum*, *L*. *acidophilus* and *Ba*. *coagulans* (p<0.05, independent t test). In addition, LPS-induced IL-10 production was enhanced by 5h and 24h fermentation supernatants from B-GOS, inulin, microcrystalline and maltodextrin (p<0.01, independent t test). It was also enhanced by 5h and 24h supernatants from *B*. *bifidum*, *L*. *acidophilus*, *Bac*. *thetaiotaomicron* and *Ba*. *coagulans* fermentations (p<0.05, independent t test). The LPS-induced IL-6 production was only enhanced by 24h supernatants from *Ba*. *coagulans* (p = 0.008, independent t test).

## Discussion

Prebiotics and probiotics have been shown to modulate the intestinal bacterial composition towards a potentially healthy composition in elderly populations in several studies [2, 13, 42, 43]. The current study directly compared the impact of both prebiotics and probiotics on the gut microbiota of elderly volunteers using an *in vitro* approach; then using an *ex vivo* approach monitored the potential impact on selected immune parameters.

In the current study B-GOS led to a positive microbial shift, with the potential for reduced inflammation by stimulating bifidobacteria growth, enhancing IL-10 production and inhibiting TNF-α production. Positive effects of B-GOS on colonic bacterial balance with stimulation of bifidobacteria, concurrent with reduced inflammation following intervention was observed by Vulevic *et al*., [13]. The reduced inflammatory potential observed in the current study was not as dramatic as that observed in the *in vivo* study of Vulevic, such differences could be related to the PBMC *in vitro* approach. The impact of B-GOS on the microbiota has been observed in different clinical settings, such as, overweight adults [52] and Irritable Bowel Syndrome patients [53]. In addition, an *in vitro* study looking at the porcine microbiota also confirmed the positive effects of B-GOS [54]. In the current study, the positive effect of B-GOS on beneficial bacteria at the expense of pathogenic bacteria showed that B-GOS intervention could lead to a potentially beneficial shift of microbiota composition in elderly persons [13, 55]. This is relevant when considering the changes that occur in the microbiota during ageing, this includes lower levels of bifidobacteria and increased inflammation. The results from the current study also showed a positive microbial shift following inulin with stimulation of bifidobacteria and lactobacilli which has also been supported by several *in vivo* and *in vitro* studies [2, 56–58].

The current study confirmed the bifidogenic effects of *B*. *bifidum* used as a probiotic, rather than in a synbiotic combination. In previous studies, synbiotics containing *B*. *bifidum* were also shown to induce a significant stimulatory effect of the bifidobacteria genus rather than *B*. *bifidum* alone [16, 59]. Furthermore, the stimulation of lactobacilli by probiotic *L*. *acidophilus* in this study was similar to that of a synbiotic containing *L*. *acidophilus*, observed to increase faecal lactobacilli levels in healthy elderly [42, 60]. This shows that both of these probiotics possess this functionality in the absence of a prebiotic.

Both prebiotics and probiotics may modulate the microbiota composition by targeting different beneficial bacterial groups. Consequently, gut barrier function may be improved, pathogen infections reduced and disease risk decreased. Prebiotics showed the potential to lead to greater microbiota modulation at the genus level compared to probiotics in the current *in vitro* study. When comparing B-GOS and inulin, B-GOS showed a greater stimulatory effect on positive bacteria and a greater inhibitory effect on harmful bacteria. This indicates that under the current conditions, B-GOS was a more effective prebiotic candidate in modulating microbiota composition.

Changes in SCFA production were associated with microbiota influences following treatment. As bifidobacteria and *E*. *rectale*–*C*. *coccoides* are producers of acetic acid [61] and butyric acid [13, 61], respectively, B-GOS and inulin showed stimulatory effects on these two acids. Alterations in SCFA and BCFA production suggest proteolytic fermentation was reduced upon fermentation of B-GOS, inulin, *B*. *bifidum*, *L*. *acidophilus* and *Ba*. *coagulans*. Proteolysis is often associated with dysbiosis and negative fermentation end-products, such as ammonia and nitrosamines [62]. As such the results indicate a potential shift in fermentation to the more beneficial saccharolysis.

Although a few studies have shown that prebiotics and probiotics could directly modulate cytokine production of elderly people *in vitro* [63–66], the current study is the first to directly compare their effects. In addition, the metabolites of pathogenic and commensal bacteria were considered. Cell-free supernatants contain batch culture medium, faecal water and metabolites of substrates. LPS would invoke an immune response and subsequently stimulate production of immune markers. Cell free fermentation metabolites may subsequently have anti-inflammatory effects by inhibiting production of TNF-α and enhancing production of IL-10. The SCFA production would become stable after 24 hours, therefore batch culture supernatants at 0h, 5h and 24h were collected and incubated with LPS and PBMC.

The down-regulation effects of metabolites from prebiotics and probiotics on TNF-α suggest anti-inflammatory potential. A positive impact may be directly associated with fermentation end products of prebiotics and probiotics. A few studies have shown that TNF-α production induced by stimuli *in vitro* could be inhibited by SCFA, especially butyrate and acetate [67–70]. This study showed fermentation supernatants from prebiotics and probiotics contained high levels of acetate and butyrate, with anti-inflammatory potential. Therefore, this study indicated the beneficial effects of prebiotics and probiotics metabolites and their beneficial effects on selected immune markers in elderly.

IL-10 is an important anti-inflammatory cytokine, which may counteract the production of proinflammatory cytokines, such as TNF-α [71, 72]. In this study, supernatants from prebiotic and probiotic fermentations enhanced production of IL-10 *in vitro*. There may be several fermentation metabolites associated with this impact, for example SCFA [67, 69, 72]. Similarly, enhancement of IL-10 production by *Bac*. *thetaiotaomicron* may be also linked to its fermentation end products, although the increase was not as dramatic as that produced by prebiotics and probiotics. In this study *S*. *typhimurium* has not been found to change inflammation status, although prebiotics and probiotics led to a more positive inflammatory status.

Prebiotics (B-GOS, and inulin) and probiotics (*B*. *bifidum*, *L*. *acidophilus* and *Ba*. *coagulans*) led to a change in the balance of the microbiota to a potentially positive balance, as seen by an increase in bifidobacteria, a group known to be at reduced levels in older people. Furthermore, supernatants from prebiotic and probiotic fermentations showed an anti-inflammatory effect by inhibiting production of pro-inflammatory cytokines and enhancing production of anti-inflammatory cytokines which was possibly related to SCFA concentrations. This research indicates that prebiotics and probiotics have huge potential for modulating the microbiota and inflammation status of elderly people. Furthermore, the prebiotic effect observed was more marked than that of probiotics. Such results are important when evaluating the best treatment to use in targeted interventions.

## Supporting Information

S1 Fig Mean bacterial populations in pH-controlled batch cultures at 0 (white), 5 (shaded), 10 (spots), 24 (fine diaganol lines), 30 (spaced diagonal lines) and 48h (black). (A) *Atopobium* cluster changes during batch culture fermentation. (B) *Bacteroides—Prevotella* spp. changes during batch culture fermentation. (C) Total bacteria changes during batch culture fermentation. Values are mean ± SD from triplicate samples.*, significant differences from the 0h value within the same treatment, p<0.05. Significant differences (p<0.05) among treatments at the same time point are indicated with different letters from the same colour of column.
(TIF)

S2 Fig SCFA concentrations in pH-controlled batch cultures at 0 (white), 5 (shaded), 10 (spots), 24 (fine diaganol lines), 30 (spaced diagonal lines) and 48h (black). (A) Propionate production during batch culture fermentation. (B) iso-Butyrate production during batch culture fermentation. (C) iso-Valerate production during batch culture fermentation. Values are mean ± SD from triplicate samples. *, significant differences from the 0h value within the same treatment, p<0.05. Significant differences (p<0.05) among treatments at the same time point are indicated with different letters from the same colour of column.
(TIF)

S3 Fig Effect of fermentation supernatants from batch cultures on cytokine production by peripheral blood mononuclear cells (PBMC). Supernatants at 0 (white), 5 (shaded) and 24h (black). Supernatants from PBMCs cultured without batch culture supernatant were used as controls (+/-) (spaced diagonal lines). (A) IL-8 production by PBMC. (B) IL-1β production by PBMC. Values are mean ± SD. PBMC from three volunteers was incubated with batch culture supernatants for 24h. #, significant differences from LPS value p<0.05. *, significant difference from 0-h value within the same fermentation treatments. At the same time point, differences among different treatments in cytokines production were analysed by one-way ANOVA. Significant differences (p<0.05) determined by post hoc Tukey HSD test were not found. In addition, cytokines in non-stimulated PBMC (blank) and in pure batch culture medium-treated PBMC (batch) were also determined. There was no significant difference between them. There was also no significant difference between LPS (LPS-stimulated PBMC) and batch+LPS (PBMC incubated with pure batch culture medium and LPS).
(TIF)
