# Supplementary figures and images for: An In Vitro Approach to Study Effects of Prebiotics and Probiotics on the Faecal Microbiota and Selected Immune Parameters Relevant to the Elderly
Source: PLoS One. 2016 Sep 9;11(9):e0162604. doi: 10.1371/journal.pone.0162604 (PMC5017648; doi:10.1371/journal.pone.0162604)

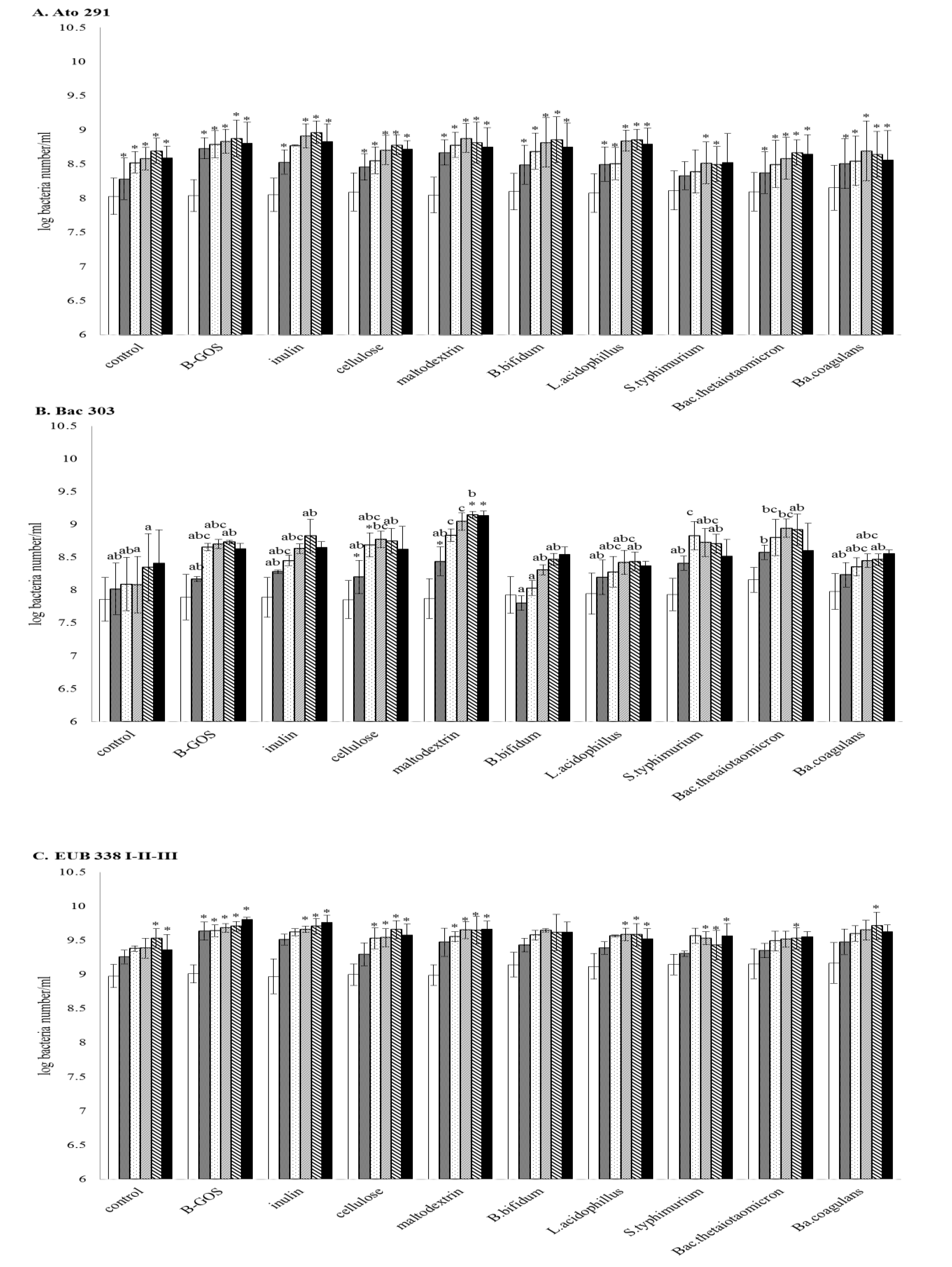

Supplement: S1 Fig — (A) Atopobium cluster changes during batch culture fermentation. (B) Bacteroides—Prevotella spp. changes during batch culture fermentation. (C) Total bacteria changes during batch culture fermentation. Values are mean ± SD from triplicate samples.*, significant differences from the 0h value within the same treatment, p<0.05. Significant differences (p<0.05) among treatments at the same time point are indicated with different letters from the same colour of column. (TIF) [file pone.0162604.s001.tif]

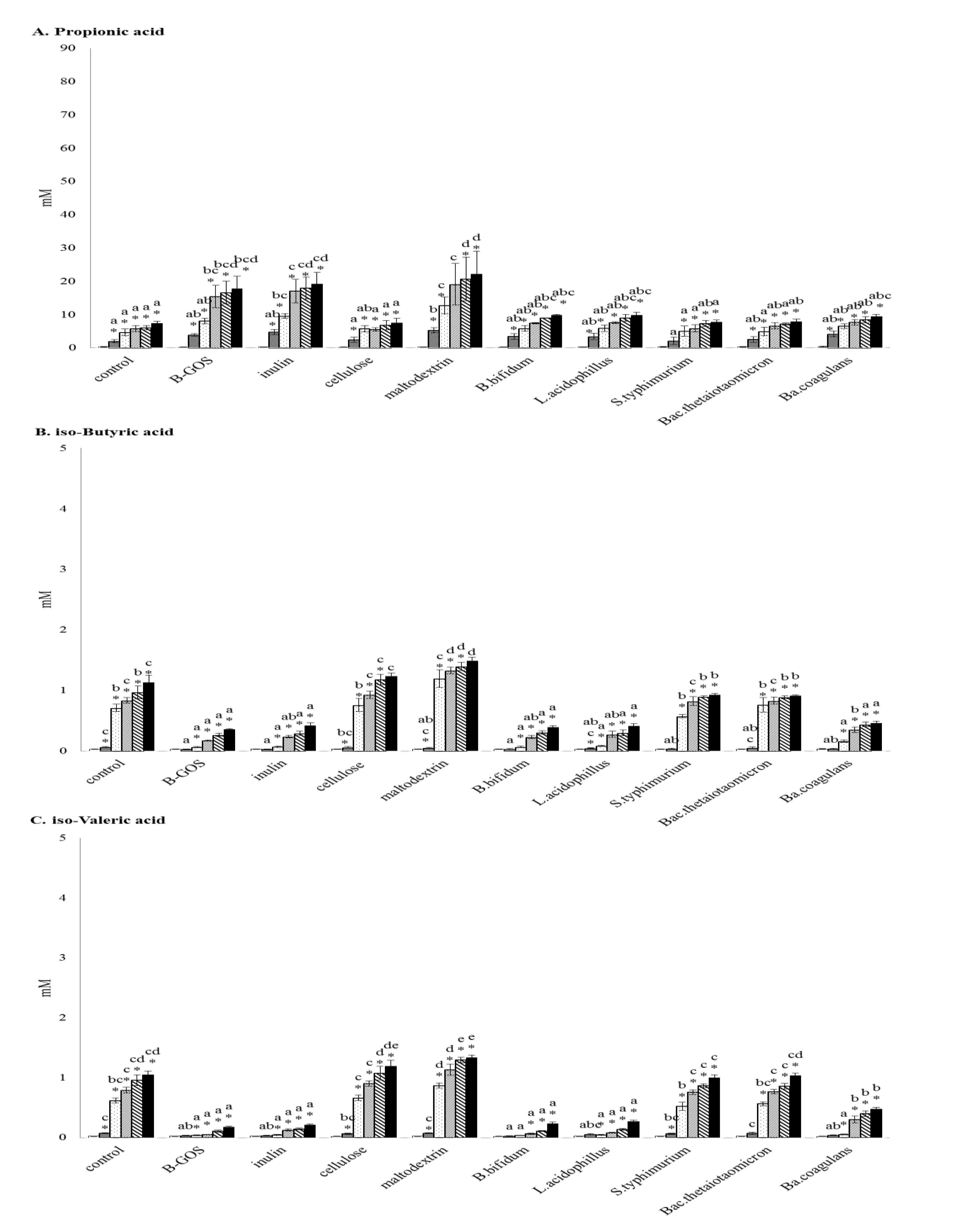

Supplement: S2 Fig — (A) Propionate production during batch culture fermentation. (B) iso-Butyrate production during batch culture fermentation. (C) iso-Valerate production during batch culture fermentation. Values are mean ± SD from triplicate samples. *, significant differences from the 0h value within the same treatment, p<0.05. Significant differences (p<0.05) among treatments at the same time point are indicated with different letters from the same colour of column. (TIF) [file pone.0162604.s002.tif]

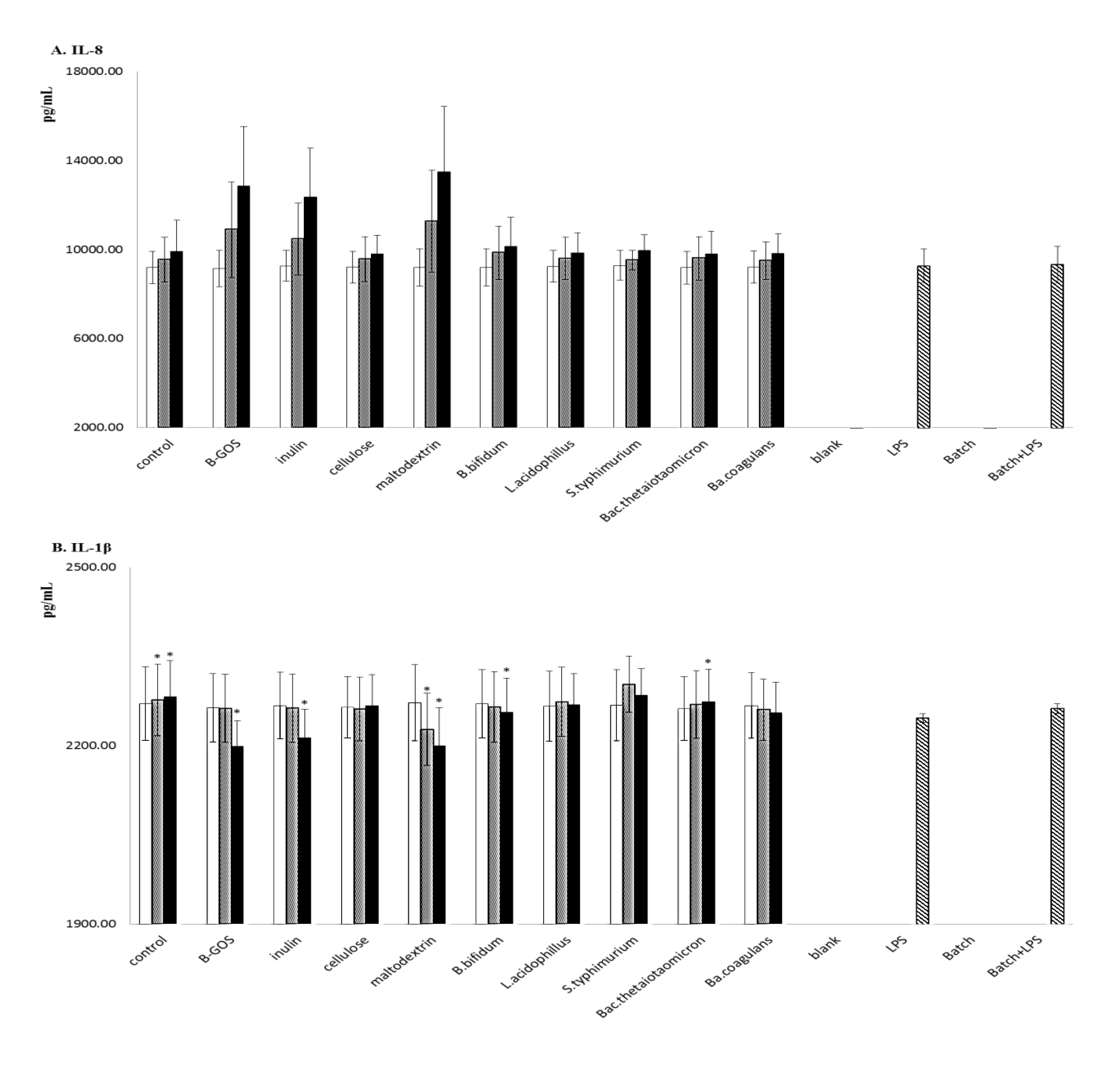

Supplement: S3 Fig — Supernatants from PBMCs cultured without batch culture supernatant were used as controls (+/-) (spaced diagonal lines). (A) IL-8 production by PBMC. (B) IL-1β production by PBMC. Values are mean ± SD. PBMC from three volunteers was incubated with batch culture supernatants for 24h. #, significant differences from LPS value p<0.05. *, significant difference from 0-h value within the same fermentation treatments. At the same time point, differences among different treatments in cytokines production were analysed by one-way ANOVA. Significant differences (p<0.05) determined by post hoc Tukey HSD test were not found. In addition, cytokines in non-stimulated PBMC (blank) and in pure batch culture medium-treated PBMC (batch) were also determined. There was no significant difference between them. There was also no significant difference between LPS (LPS-stimulated PBMC) and batch+LPS (PBMC incubated with pure batch culture medium and LPS). (TIF) [file pone.0162604.s003.tif]
